# Fourteen genome sequences from bacterial, environmental isolates from the Cotton Glacier stream, Antarctica

**DOI:** 10.1128/mra.00133-25

**Published:** 2025-08-13

**Authors:** Christine M. Foreman, Heidi J. Smith, Markus Dieser

**Affiliations:** 1 Center for Biofilm Engineering, Montana State University 827241, Bozeman, Montana, USA; 2 Department of Chemical & Biological Engineering, Montana State University 33052, Bozeman, Montana, USA; 3 Department of Microbiology and Cell Biology, Montana State University 123776, Bozeman, Montana, USA; DOE Joint Genome Institute, Berkeley, California, USA

**Keywords:** supraglacial, Antarctica, biofilm

## Abstract

We report the genomic sequences of 14 bacterial isolates from a supraglacial stream on the Cotton Glacier, Antarctica. Fine sediments in the streambed provide habitat for bacterial growth and biofilm formation. The stream represents a natural laboratory for studying the evolution and adaptation of microbes to a humic-free environment.

## ANNOUNCEMENT

The Cotton Glacier stream (77°07′S, 161°40′E), Antarctica, is a ~16 km long sediment-laden, supraglacial stream. Detailed studies have highlighted its biogeochemical processes and downstream ramifications (1–5). This stream represents a natural laboratory for studying the evolution and adaptation of microbes to the absence of humics (1). Stream water was collected during the austral summers of 2009 and is described elsewhere (1). Bacteria were isolated aerobically on R2A agar plates at 4°C in the dark. Isolates were selected and cultured based on colony morphology. Isolates stored in 25% glycerol at –80°C were grown on R2A agar plates at 4°C for 7 days. Single colonies were then inoculated in R2A broth (20 mL) at 4°C while shaking at 150 rpm to the late exponential phase. The cetyltrimethylammonium bromide procedure was used for extracting DNA (6).

Standard quality shotgun libraries for bacterial strains CG_S6, CG_S7, CG_23.3, CG_9.1, CG_9.2, CG_9.5, CG_9.7, CG_9.8, CG_9.10, and CG_9.11 were sequenced on the Illumina NovaSeq S4 platform (2 × 151 bp paired-end reads) (7). Libraries were prepared on the PerkinElmer Sciclone NGS robotic liquid handling workstation using the Kapa Biosystems Library Kit. DNA was sheared to 459 bp using a Covaris LE220 focused-ultrasonicator. DNA fragments were size-selected by double-SPRI. Fragments were end-repaired, A-tailed, and ligated with Illumina-compatible sequencing adaptors. Raw Illumina sequences were quality filtered using BBTools (8) per JGI SOP 1061 and assembled with SPAdes (≥version v3.14.1; –phred-offset 33 –cov-cutoff auto -t 16 m 64 –careful -k 25,55,95) (9). Contigs with a length <1 kb (BBTools ≥ v.38.86, reformat.sh: minlength = 1,000 ow = t) were discarded.

For improved high-quality draft genomes, PacBio SMRTbell libraries for bacterial strains CG_23.4, CG_23.5, CG_23.6, and CG_9.6 were sequenced on the Pacific Biosciences (PacBio) Sequel II platform (10). DNA was sheared around 10 kb using Megaruptor 3 (Diagenode). Sheared DNA was treated with exonuclease, DNA repair enzyme mix, end-repair/A-tailing mix, and ligated with barcoded overhang adapters using SMRTbell Express Template Prep Kit 2.0 (PacBio). Libraries were purified with AMPure PB Beads (PacBio) and bound to Sequel II polymerase 2.0 using the Sequel II Binding Kit 2.0. Libraries were sequenced using tbd-sample dependent sequencing primers, 8M v1 SMRT cells, and Version 2.0 sequencing chemistry with 1 × 900 sequencing movie run times. Additionally, collapsed adapters were removed with BBTools v38.87. Reads >5 kb from CG_23.5 and CG_9.6 were assembled with the hierarchical genome assembly process [HGAP] (smrtlink/8.0.0.80529, HGAP 4 (1.0)) using default settings (11). Sequence reads from isolates CG_23.4 and CG_9.6 were assembled with Flye 2.8.1 (–asm-coverage 100 –plasmid –genome-size 5000000 t 64 –pacbio-raw (fasta list)) (12). Contigs with a probability of being a plasmid were identified using TensorFlow (13).

CRISPR elements were identified using the programs CRT v.1.8.2 (CRISPR Recognition Tool) (14) or PILER-CR (15). All genomes were annotated in the Integrated Microbial Genomes (IMG) database (≥IMG Annotation Pipeline v.5.1.2) (16). Genome completeness was estimated using checkM v.1.2.2 (17). The genome-sequence-acquired 16S ribosomal RNA gene was queried in command line against the SILVA database 138.1 with blastn, from BLAST+2.13.0 for taxonomic classification (18). Sequence details are given in Table 1.

**TABLE 1:** Summary of genome characteristics^*a*^^,^^*b*^

Platform	Organism [% match to genus, accession #]] Accession number	# reads Coverage (×) Completeness (%) Contamination (%)	# Contigs	*L*_50_/*N*_50_ (bp)	Size (bp)	GC (%)	# Genes	# C	# P
Illumina	*Janthinobacterium* sp. CG_S6 [98.1; NR_132608.1] JAXCHS000000000 SRR25764247	13,218,236 246.4 99.26 0.19	79	12/181,542	6,110,077	65.69	5,505	2	–
Illumina	*Pedobacter* sp. CG_S7 [98.2; NR_148294.1] JBEONS000000000 SRR28961170	1,691,946 405.4 99.84 0.96	108	12/93,633	3,854,172	37.16	3,582	2	–
Illumina	*Janthinobacterium* sp. CG_23.3 [98.1; NR_132608.1] JBJXIB000000000 SRR30853869	8,213,898 256.4 98.46 0.35	280	51/38,360	6,116,288	65.55	5,722	2	–
PACBIO	*Janthinobacterium* sp. CG_23.4 [99,4; NR_132608.1] JARXUX000000000 SRR12927869	905,709 193.2 99.95 0.0	2	1/4,988,477	5,012,930	60.74	4,596	–	1
PACBIO	*Flavobacterium* sp. CG_23.5 [98.4; NR_043891.1] JAGGNA000000000 SRR12927877	396,643 261.4 98.94 0.0	1	1/3,672,252	3,672,252	33.93	3,365	1	–
PACBIO	*Polaromonas* sp. CG_23.6 [99.2; NR_109013.1] JARXUY000000000 SRR12927893	637,596 214.6 99.66 0.25	4	1/4,099,181	4,511,363	60.01	4,444	–	3
Illumina	*Flavobacterium* sp. CG_9.1 [99.8; NR_113714.1] JADOVC000000000 SRR13164898	5,107,940 201.4 98.94 2.68	86	12/111,844	3,938,215	34.35	3,644	1	–
Illumina	*Polaromonas* sp. CG_9.2 [99.2; NR_109013.1] JADOVF000000000 SRR13164963	8,771,292 323.6 99.66 0.25	55	8/192,115	4,152,851	60.1	4,012	–	–
Illumina	*Polaromonas* sp. CG_9.5 [99.3; NR_109013.1] JAXCHR000000000 SRR25764248	6,678,012 390.8 99.73 0.0	76	9/157,676	3,803,867	60.18	3,659	–	–
PACBIO	*Cryobacterium* sp. CG_9.6 [99.3; NR_042170.1] JARXUZ000000000 SRR12927894	659,510 271.1 99.24 0.51	2	1/3,352,313	3,539,656	63.32	3,434	2	1
Illumina	*Polaromonas* sp. CG_9.7 [99.2; NR_109013.1] JADOVG000000000 SRR13164962	1,787,966 66.5 99.66 0.25	55	8/192,115	4,152,641	60.1	4,007	–	–
Illumina	*Frigoribacterium* sp. CG_9.8 [97.9; NR_026511.1 JADOVH000000000 SRR13164979	9,006,494 475.3 99.49 0.51	11	2/614,762	2,845,437	63.3	2,825	1	–
Illumina	*Flavobacterium* sp. CG_9.10 [99.3; NR_199451.1] JADOVD000000000 SRR13164902	4,286,438 191.7 98.94 0.0	43	7/210,281	3,529,796	33.79	3,284	1	–
Illumina	*Polaromonas* sp. CG_9.11 [99.2; NR_109013.1] JADOVE000000000 SRR13164964	7,279,460 270.2 99.61 0.03	77	10/145,329	4,267,931	61.41	4,191	–	–

^*a*^ C = CRISPR, P = Plasmid.

^*b*^ –, none.

## Data Availability

The work conducted by the U.S. Department of Energy Joint Genome Institute, a DOE Office of Science User Facility, is supported under Contract No. DE-AC02-05CH11231. These data were generated for JGI Proposal #505037. The genome sequences have been submitted to GenBank and are available through the accession numbers listed in Table 1.
